# Author Correction: TRPS1 shapes YAP/TEAD-dependent transcription in breast cancer cells

**DOI:** 10.1038/s41467-018-06266-2

**Published:** 2018-09-12

**Authors:** Dana Elster, Marie Tollot, Karin Schlegelmilch, Alessandro Ori, Andreas Rosenwald, Erik Sahai, Björn von Eyss

**Affiliations:** 1 Leibniz Institute on Aging, Fritz Lipmann Institute e.V., Beutenbergstr. 11, 07745 Jena, Germany; 2 Tumour Cell Biology Laboratory, Francis Crick Institute, 1 Midland Road, London, NW1 1AT UK; 3 Institute of Pathology, University of Würzburg, and Comprehensive Cancer Center Mainfranken (CCCMF), Josef-Schneider-Str. 2, 97080 Würzburg, Germany

Correction to: *Nature Communications*; 10.1038/s41467-018-05370-7; published online 6 August 2018

In the original version of this article, financial support was not fully acknowledged. The PDF and HTML versions of the Article have now been corrected to include the following:

“This work was supported by the Francis Crick Institute, which receives its core funding from Cancer Research UK (FC001144), the UK Medical Research Council (FC001144), and the Wellcome Trust (FC001144).”

