# Supplementary material for: TRPS1 shapes YAP/TEAD-dependent transcription in breast cancer cells
Source: Nat Commun. 2018 Aug 6;9:3115. doi: 10.1038/s41467-018-05370-7 (PMC6079100; doi:10.1038/s41467-018-05370-7)
Supplement: Supplementary file 1 — Supplementary Information [file 41467_2018_5370_MOESM1_ESM.pdf]

Supplementary Information

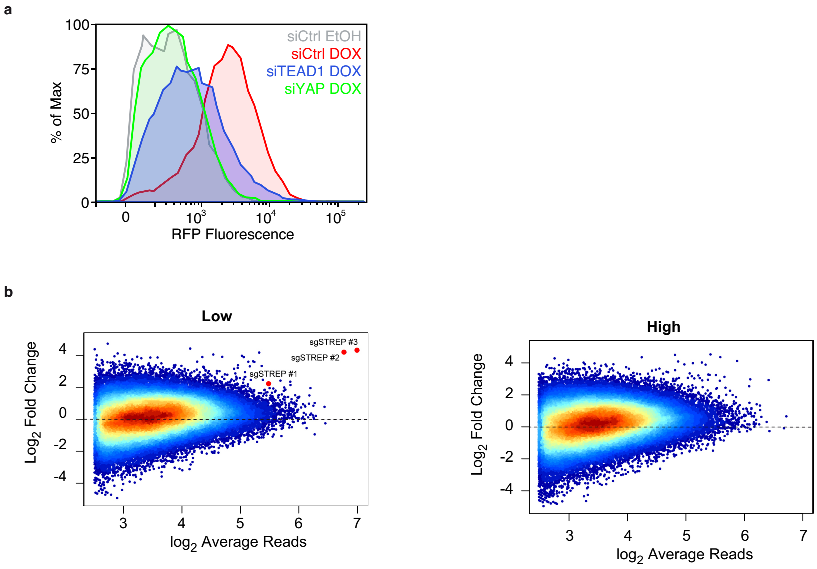

Supplementary Figure 1
(a) Flow cytometry for RFP in doxycycline-induced sensor cells transfected with a control siRNA (siCtrl) or siRNAs targeting TEAD1 or YAP, respectively. (b) sgRNAs enrichment, as determined by Next-Generation Sequencing, in Low *vs* unsorted cells (left) and in High *vs* unsorted cells (right) after infection of the sensor cells with the GeCKO v2 lentiviral CRISPR library.

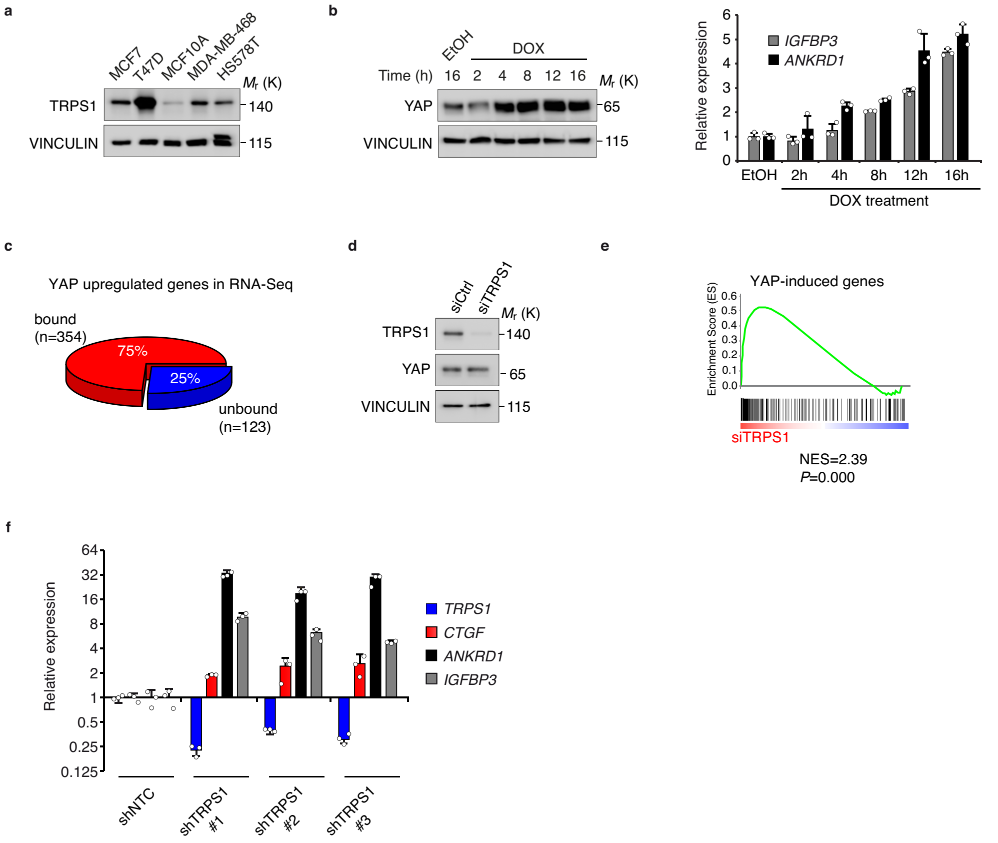

Supplementary Figure 2

(a) Western blot for TRPS1 in different breast cancer cell lines. Equal amounts of lysates were loaded per cell line. Vinculin serves as loading control.

(b) Western blot for YAP (left) and qRT-PCR analysis of *IGFBP3* and *ANKRD1* expression (right) at different time points after doxycycline induction (DOX) of Strep-YAP 5SA expression in MCF7 i5SA cells. Ethanol (EtOH) was used as uninducing control. Error bars represent s.d..

(c) Pie chart depicting the proportion of YAP bound (direct targets) and unbound (indirect targets) promoter regions among the genes that were upregulated in RNA-Seq.

(d) Western blot for TRPS1 and YAP after depletion of TRPS1 in T47D cells by siRNAs. siCtrl: siRNA control. Vinculin serves as loading control.

(e) Gene set enrichment analysis (GSEA) for RNA-Sequencing from TRPS1-depleted MCF7 cells for a set of 497 YAP-induced genes. NES: Normalized Enriched Score.

(f) qRT-PCR analysis of the expression of *TRPS1*, *CTGF*, *ANKRD1* and *IGFBP3* after shRNA-mediated depletion of TRPS1 in MCF7 cells. shNTC: shRNA non-targeting control. Error bars represent s.d..

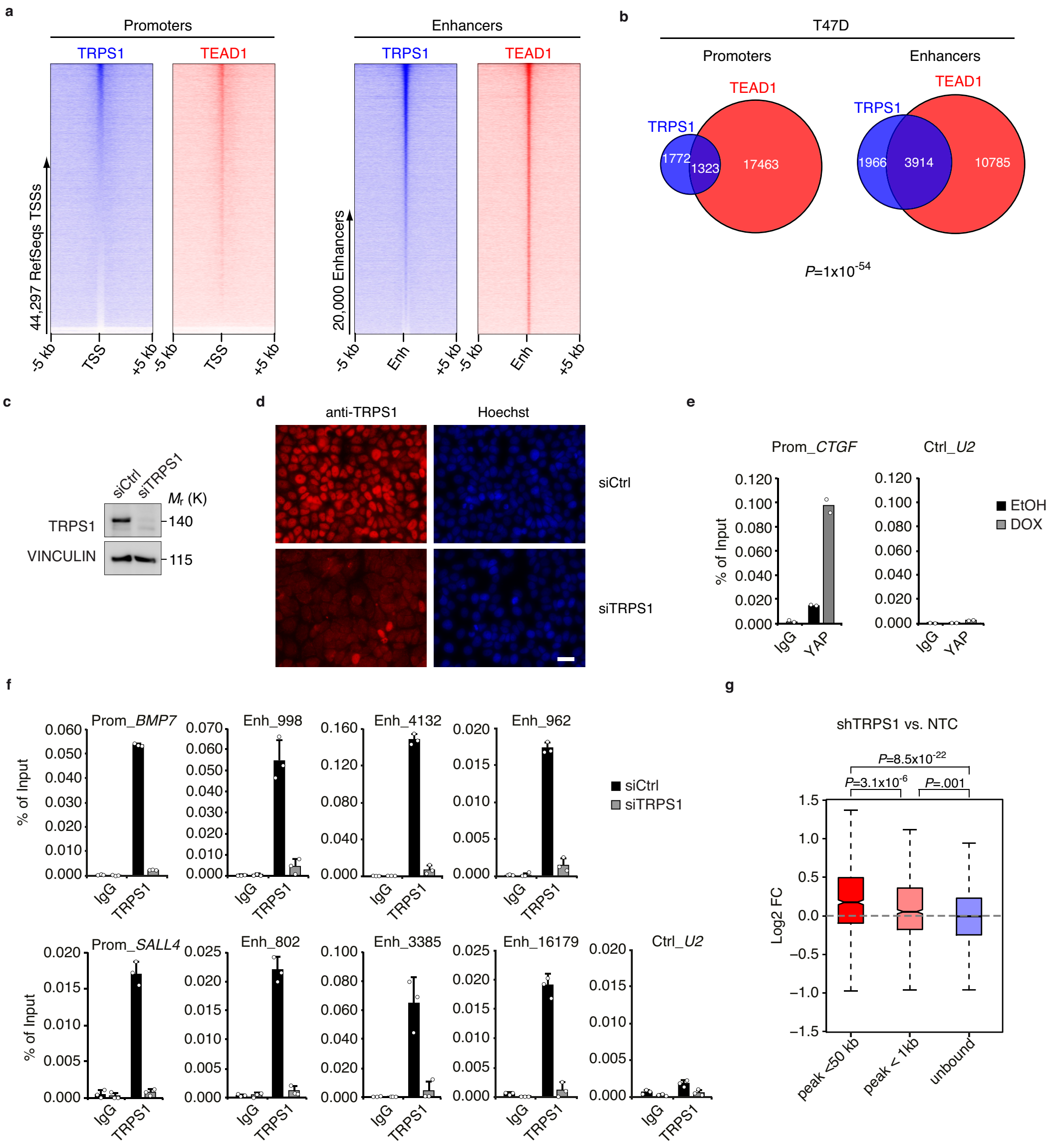

Supplementary Figure 3

(a) Heatmaps of ChIP-Seq data from T47D cells showing the occupancy of TRPS1 and TEAD1 at all RefSeq transcriptional start sites (TSSs) and all enhancer regions. The heatmaps were sorted according to TRPS1 binding.

(b) Venn diagram showing the overlap of enhancer sites bound by TRPS1 and TEAD1 in T47D cells.

(c) Western blot for TRPS1 in MCF7 cells transfected with siCtrl or siRNAs targeting TRPS1.

(d) Immunofluorescence stainings for TRPS1 in MCF7 cells with the TRPS1-specific antibody generated for ChIP experiments. Cells were transfected with either siTRPS1 or siCtrl. Hoechst counterstaining was used to stain nuclei. Scale bar = 50 µm.

(e) qChIP analysis for YAP binding at the *CTGF* promoter in MCF7 i5SA cells after induction by doxycycline (DOX) or ethanol control (EtOH). *U2* serves as control region. Control IPs were performed using rabbit IgG.

(f) qChIP analysis for TRPS1 binding at two promoters (Prom) and 6 different enhancer sites (Enh) in MCF7 cells transfected with siCtrl or siRNA targeting

TRPS1. *U2* serves as control region. Control IPs were performed using rabbit IgG. Error bars represent s.d..
(g) Boxplot for the expression changes after TRPS1 depletion (shTRPS1 vs. shNTC). The genes were stratified according to the presence of a TRPS1 ChIP-Seq peak in a 50 kb window (left boxplot), a peak in a 1 kb window (middle boxplot) and having no TRPS1 peak (right boxplot) in a 50 kb window. For both "bound" groups (50 kb and 1 kb) the 1000 peaks with the highest confidence (based on FDR) were selected for this analysis. NTC = non-targeting control; Log2 FC = Log2 fold change. Median: black line; boxes: data points between the first and third quartiles; whiskers: up to 1.5x interquartile range; points: outliers. Wilcox-test, two-sided.

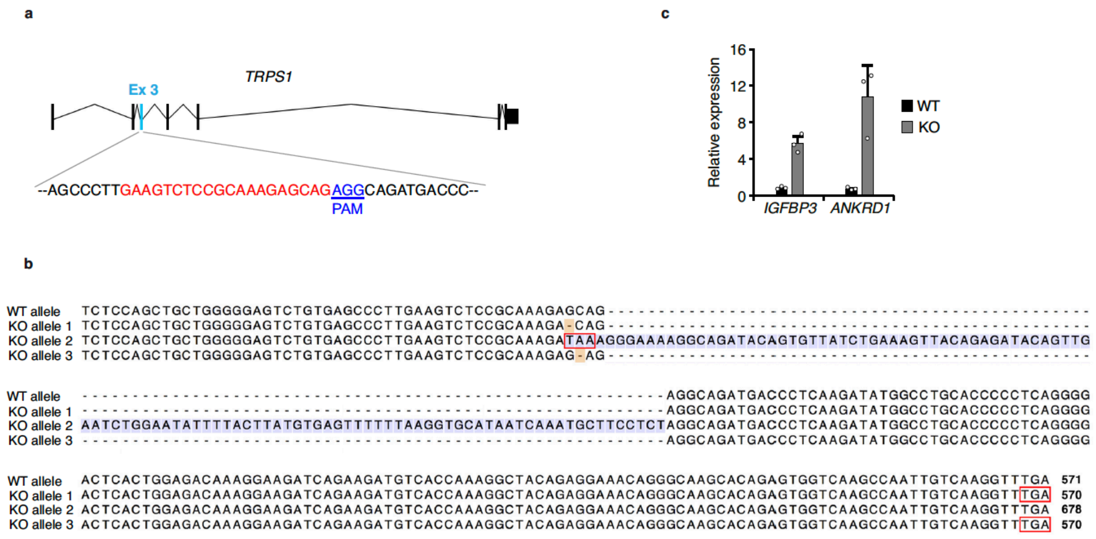

Supplementary Figure 4

(a) Schematic of the *TRPS1* locus. The position of the sequence targeted by the sgRNA in exon 3 (Ex3) is indicated in red. This sgRNA was used for generation of TRPS1 CRISPR KO cells.

(b) Alignment of the three different KO alleles identified by Sanger sequencing after CRISPR-mediated genome editing of the *TRPS1* locus. The WT allele is given at the top. Insertion and deletions are highlighted in blue and orange, respectively. Newly created premature stop codons are shown with red boxes.

(c) qRT-PCR analysis of *IGFBP3* and *ANKRD1* expression in MCF7 WT *vs* TRPS1 KO. The data summarizes three technical replicates. Error bars represent s.d..

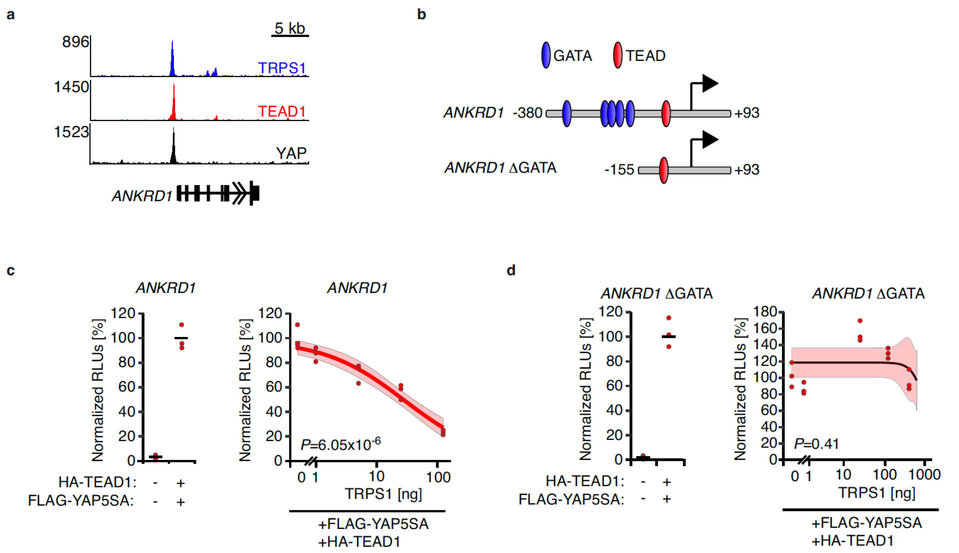

Supplementary Figure 5
(a) ChIP-Seq tracks for TRPS1, TEAD1 and YAP at the *ANKRD1* transcription start site.
(b) Schematic of the two *ANKRD1* promoter versions used to drive the luciferase reporter: the full-length sequence (*ANKRD1*) and a truncated version lacking the GATA sites (*ANKRD1ΔGATA*).
(c) Luciferase activity of the reporter driven by *ANKRD1* in 293T cells co-transfected with vectors for expression of FLAG-YAP 5SA and HA-TEAD1. The left chart shows the luciferase reporter activity induced by TEAD1/YAP 5SA in the absence of TRPS1. The right chart shows the repressing effect of increasing amounts of TRPS1 on the reporter activity as a dose response curve. Data presented are from 3 biological replicates. The *P*-value describes if increasing TRPS1 concentrations affect the luciferase signal, one-way ANOVA.
(d) Luciferase activity of the reporter driven by *ANKRD1* DGATA in 293T cells co-transfected with vectors for expression of FLAG-YAP 5SA and HA-TEAD1. The left chart shows the luciferase reporter activity induced by TEAD1 in the absence of TRPS1. The right chart shows the repressing effect of increasing amounts of TRPS1 on the reporter activity. Data presented are from three biological replicates. The *P*-value describes if increasing TRPS1 concentrations affect the luciferase signal, one-way ANOVA. Normalized RLUs: Relative luciferase light units.

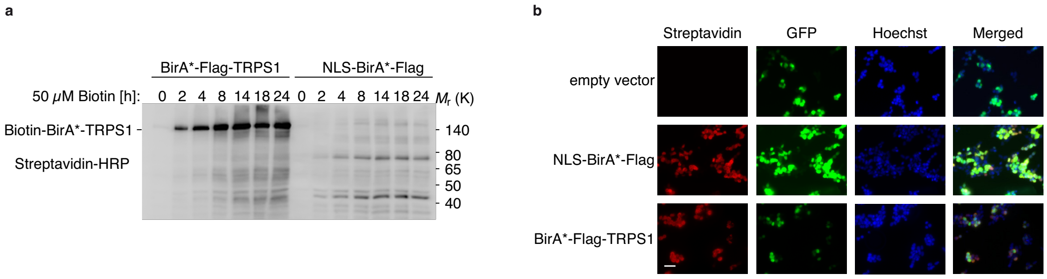

Supplementary Figure 6

(a) Western blot for proteins biotinylated by BirA*-Flag-TRPS1 and NLS-BirA*-Flag at different time points after addition of biotin. Biotinylated proteins were detected by streptavidin coupled to horseradish peroxidase (Streptavidin-HRP).

(b) Immunofluorescence stainings of 293T cells transfected with NLS-BirA*-Flag or BirA*-Flag-TRPS1 constructs or an empty vector control. Biotinylated proteins were identified by Streptavidin PE-Cy7. Transfected cells were identified by GFP that is co-expressed by means of an IRES element. Hoechst is used to stain nuclei. Scale bar = 50 μm.

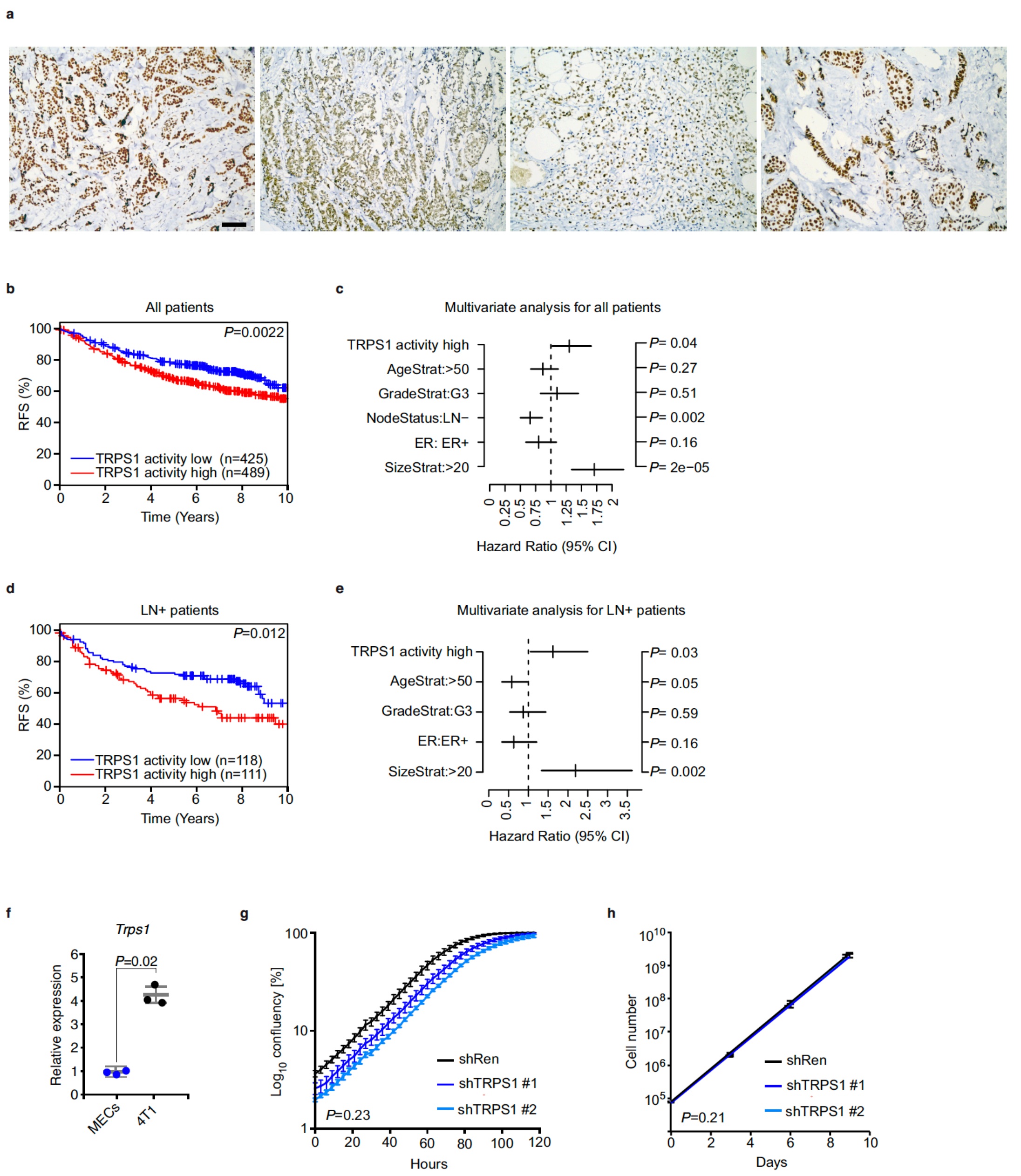

Supplementary Figure 7

(a) Immunohistochemical staining for TRPS1 on tissue sections from four representative human breast cancer patients. Scale bar = 100 µm.

(b) Kaplan-Meier plot for the survival probability of breast cancer patients that were stratified according to their TRPS1 activity. TRPS1 activity was defined by the expression of a TRPS1-repressed target gene set. High TRPS1 activity is consequently associated with low expression of this gene set. The *P*-values were determined by a Chi-square test. RFS: recurrence-free survival.

(c) Multivariate analysis including the given parameters for patients included in (b). The hazard ratio and the corresponding 95% confidence interval (CI) are given as a vertical or horizontal line, respectively.

(d) The same analysis as in (b) was performed, except that only lymph node-positive (LN+) patients were included in the analysis. RFS: recurrence-free survival.

(e) Multivariate analysis including the given parameters for patients included in (d). The hazard ratio and the corresponding 95% confidence interval (CI) are given as a vertical or horizontal line, respectively.
(f) qRT-PCR analysis for *Trps1* expression in FACS-purified mouse mammary epithelial cells (MECs) and 4T1 cells, respectively. Each dot represents a biological replicate. The *P*-Value was determined by a Welch's t-test.
(g) Incucyte growth rate of 4T1 cells infected with a control shRenilla or two different shRNAs targeting Trps1. A one-way ANOVA test was performed to test for differences in doubling time.
(h) Cumulative growth curve of 4T1 cells infected with a control shRenilla or two different shRNAs targeting Trps1. A one-way ANOVA test was performed to test for differences in doubling time.

Supplementary Table 1 – Antibodies

Application	Antibody	Company	Reference	Dilutions
Western Blot	anti-YAP	Cell signaling	14074	1:1000
	anti-CTGF	Santa Cruz	sc-14939	1:500
	anti-Vinculin	Sigma-Aldrich	V9131	1:100000
	anti-TRPS1	Abcam	ab209664	1:2000
	anti-FLAG	Sigma-Aldrich	F1804	1:2000
	anti-HA	Covance/ Biolegend	901502 / B220767	1:2000
	anti-GAL4	Santa Cruz	sc-510	1:2000
	anti-TRPS1	Bethyl	A303-563A	1:2000
	anti-V5	Cell signaling	13202S	1:2000
	anti-CTBP2	BD Bioscience	612044	1:1000
	anti-SMRT	Millipore	06-891	1:1000
	anti-HDAC1	Gift from F. Neri	-	1:1500
	anti-Pan-TEAD	Cell signaling	13295	1:2000
	anti-rabbit IgG HRP	Santa Cruz	sc-2004	1:10000
	anti-mouse IgG HRP	Santa Cruz	sc-2005	1:10000
	anti-goat IgG HRP	Santa Cruz	sc-2020	1:10000
	anti-rabbit IgG HRP	Rockland	18-8816-33	1:4000
	anti-mouse IgG HRP	Rockland	18-8817-33	1:4000
ChIP/ChIP-Seq	anti-TRPS1	selfmade	-	2 µg/10 µg
	anti-TEAD1	BD Bioscience	610923	2 µg/10 µg
	anti-YAP	Abcam	ab52771	-/10 µg
	anti-H3K27ac	Abcam	ab4729	-/5 µg
	anti-CTBP2	BD Bioscience	612044	2 µg/5 µg
	anti-HDAC3	Cell signaling	85057	5 µl /15 µl
	anti-SMRT			2 µg/-
	anti-IgG rabbit	Sigma	I5006	2 µg/5-10 µg
	anti-IgG mouse	Sigma	I5381	2 µg/5-10 µg
Co-IP exogenous/endogenous	anti-TRPS1	selfmade	-	0.75 µg/4 µg
	anti-IgG rabbit	Sigma	I5006	0.75 µg/4 µg
IF	anti-TRPS1	selfmade	-	1:400
	Streptavidin-PE-Cy7	eBioscience	25-4317-82	1:400
	anti-rabbit Alexa 546	Life Technologies	A10040	1:1000
	Hoechst 33342	Sigma	B2261	1:5000
IHC	anti-CD3 (Gamma Chain)	Abcam	ab134096	1:375
	anti-TRPS1	Abcam	ab209664	1:8000
FACS	Anti-CD31 biotin	eBiosciences	13-0311-81	1:50
	Anti-CD45 biotin	eBiosciences	13-0451-85	1:50
	Streptavidin-APC	eBiosciences	17-4317-82	1:200
	Anti-EpCAM-PE	eBiosciences	12-5791-81	1:100
	Anti-CD49f-FITC	eBiosciences	11-0495-80	1:100

Supplementary Table 2 - Primers

Application	Primer	Fw	Rev
qRT-PCR	ANKRD1	AGTAGAGGAACTGGTCACTGG	TGTTTCTCGCTTTTCCACTGTT
	b2M	GTGCTCGCGCTACTCTCTC	GTCAACTTCAATGTCGGAT
	CTGF	AATGCTGCGAGGAGTGGGTG	TTGGGTCTGGGCCAAACGTG
	IGFBP3	AGAGCACAGATACCCAGAACT	GGTGATTCAGTGTGTCTTCCATT
	NT5E	CCAGTACCAGGGCACTATCTG	TGGCTCGATCAGTCCTTCCA
	RASSF2	AAGAAGACGAGTTCATTGTGGAG	GAATGCGTTCGTTGTCATCC
	TGFB2	CAGCACACTCGATATGGACCA	CCTCGGGCTCAGGATAGTCT
	TRPS1	ATGACACTCCTGTTGGGTACT	CGTGCTGCTTGCCATAATGTT
	VGLL1	TCAGAGTGAAGGTGTGATGCT	GCACGGTTTGTGACAGGTACT
qChIP	Prom_BMP7	GGCGTGAGTTTGCTGTCTTG	TCCGAGATAACACACCCCGA
	Prom_CTGF	ATATGAATCAGGAGTGGTGCGA	CAACTCACACCGGATTGATCC
	Prom_SALL4	GGATTCCTGTTCACAAAGACTGG	TGCTTGCTTATCATTTGCCATT
	Enh_802	ACACTGTTCTTTATCGAGCCCT	TGGACTGTTGGACGGAATGAG
	Enh_962	ACTCCCTGGCACTGAGGTTA	GTGAGAAGGGAGGTGGGAGA
	Enh_998	CACCACTGCCACCTACTTCC	ACAACACCTTGCCAGACACT
	Enh_3385	TCCAGGGCACAGAGAAGAGA	TCCCCTCGAGACTTGTGGAT
	Enh_4132	CTGCTTTCCAAGGGCTCTCC	TGAGTGCATTGTGACCATGC
	Enh_16179	TCTTTGGAATGCTGCCTGACT	GGCCCCTATAGCACAATGGT
	Enh_55	AGCTCTGCCATCTTGTAACTGT	AGGAAGCATTGGTCAACAGGT
	Enh_905	CACTATTGACATTTGGTGCCAGA	ACATAAGGAGGCAGCTTGAGG
	U2_Ctrl	TTTGCTCCCACTGCCGTC	CTGAGTCTTTCGGTGCCC
4C-Seq	IGFBP3 TSS	AATGATACGGCGACCACCGAAC ACTCTTTCCCTACACGACGCTCT TCCGATCTGAGAAATAAAGTTCC TGCCTTG*C	CAAGCAGAAGACGGCATACGACT TCCTTGGTCCAAATCCG*C
	Ctr #1	AATGATACGGCGACCACCGAAC ACTCTTTCCCTACACGACGCTCT TCCGATCTGCCTCAATAAATGCA GCT*A	CAAGCAGAAGACGGCATACGAAC TGCATAGACAGTCCCTG*A
	Ctr #3	AATGATACGGCGACCACCGAAC ACTCTTTCCCTACACGACGCTCT TCCGATCTGGCCTCCATGCCTCT TTT*T	CAAGCAGAAGACGGCATACGAGC ACACTCACCACACTCAC*A
CRISPR Screen	F1_#1 GECKO Fw	AATGGACTATCATATGCTTACCGTAACTTGAAAGTATTTCG	
	R1_#3 GECKO Rev	TCTCTAGGCACCGGATCAATTGCC	
	F2_#1 GECKO TGACCA Fw	AATGATACGGCGACCACCGAGATGACCACGAGCTCTTGTGGAAAGGAC GAAACACCG	
	F2_#2 GECKO ACAGTG Fw	AATGATACGGCGACCACCGAGAACAGTGCGAGCTCTTGTGGAAAGGAC GAAACACCG	
	F2_#3 GECKO GCCAAT Fw	AATGATACGGCGACCACCGAGAGCCAATCGAGCTCTTGTGGAAAGGAC GAAACACCG	
	F2_#4 GECKO CTTGTA Fw	AATGATACGGCGACCACCGAGACTTGTACGAGCTCTTGTGGAAAGGAC GAAACACCG	

Supplementary Table 3 - shRNAs

Gene target	shRNA ID	No.	97mer oligo
TRPS1	TRPS1.1808	#1	TGCTGTTGACAGTGAGCGACAGGACAAGATAACAGTCAAATAGTGAAG CCACAGATGTATTTGACTGTTATCTTGTCCTGCTGCCTACTGCCTCGGA
TRPS1	TRPS1.758	#2	TGCTGTTGACAGTGAGCGCCAGAGTGATGCTGCAGAACTATAGTGAAG CCACAGATGTATAGTTCTGCAGCATCACTCTGATGCCTACTGCCTCGGA
TRPS1	TRPS1.4226	#3	TGCTGTTGACAGTGAGCGAAAAGTTGATAGAAGTACTCAATAGTGAAGC CACAGATGTATTGAGTACTTCTATCAACTTTCTGCCTACTGCCTCGGA

Supplementary Table 4 – siRNAs

Gene target	Dharmacon ID	Specification
NTC	D-001810-10	ON-TARGETplus Control Pool, Non-Targeting pool
TRPS1	L-009644-00	ON-TARGETplus SMART Pool, human TRPS1
YAP1	L-012200-00	ON-TARGETplus SMART Pool, human YAP1
TEAD1	L-012603-00	ON-TARGETplus SMART Pool, human TEAD1

Supplementary Table 5 - Plasmids

Plasmid	Backbone	Insert
LeGO-iG2-Puro-CTGF-turboRFP sensor	LeGO-iG2-Puro	~200 bp CTGF promoter fragment driving turboRFP
pInducer21-YAP5SA	pInducer21	YAP 5SA
pGIPZ-shTRPS1#1-3	pGIPZ (Thermo)	shTRPS1#1-3
pLeGo-EF1a-V5-TRPS1	LeGO-iG2-puro	V5-TRPS1
pCMX-Gal4-TEAD1	pCMX	Gal4-TEAD1
pCMX-Gal4-TEAD2	pCMX	Gal4-TEAD2
pCMX-Gal4-TEAD3	pCMX	Gal4-TEAD3
pCMX-Gal4-TEAD4	pCMX	Gal4-TEAD4
pCMV-2x-flag-YAP-5SA	pCDNA3	YAP5SA
pCDNA3-HA-TEAD1	pCDNA3	HA-TEAD1
pCDNA3-HA-TEAD4	pCDNA3	HA-TEAD4
pGL4.20-ANKRD1	pGL4.20	ANKRD1
pGL4.20 ANKRD1 ΔGATA	pGL4.20	ANKRD1 ΔGATA
pCMV-beta-Gal	pCMV	Beta-gal
LeGO-EF1-NLS-BirA*-Flag	LeGO-iG2-Puro	NLS-BirA*-Flag
LeGO- EF1-BirA*-Flag-V5-TRPS1	LeGO-iG2-Puro	BirA*-Flag-V5-TRPS1
pSpCas9n(BB)-2A-GFP (PX461)-Cas9 WT	pX461	sgRNA targeting TRPS1
pRL_CMV (Promega)	-	Renilla
pGL4.23_Enh998	pGL4.23	Enhancer#998
LeGO-iG2-Puro-2xflag-YAP 5SA ORF	LeGO-iG2-puro	2xflag YAP 5SA

The lentiCRISPR v2 library (Addgene # 52961) was a kind gift from Feng Zhang
LeGO-iG2 was a gift from Boris Fehse (Addgene plasmid # 27341)
pSpCas9n(BB)-2A-GFP (PX461) was a gift from Feng Zhang (Addgene plasmid # 48140)
pCMV-flag YAP2 5SA was a gift from Kunliang Guan (Addgene plasmid # 27371)
psPAX2 and pMD2.G was a gift from Didier Trono (Addgene plasmid #12259, #12260)
pCMX-Gal4-TEAD1/2/3/4 was a gift from Kunliang Guan (Addgene plasmid # 33108 / #33107 / #33106 / #33105)
pInducer21 was a kind gift from Stefan Gaubatz
