# Supplementary material for: TRPS1 shapes YAP/TEAD-dependent transcription in breast cancer cells
Source: Nat Commun. 2018 Aug 6;9:3115. doi: 10.1038/s41467-018-05370-7 (PMC6079100; doi:10.1038/s41467-018-05370-7)
Supplement: Supplementary file 3 — Description of Additional Supplementary Files [file 41467_2018_5370_MOESM3_ESM.pdf]

Description of Additional Supplementary Files

File Name: Supplementary Data 1
Description: Full multivariate analysis of breast cancer patients (stratified for TRPS1 target gene expression).
